# *Melampyrum sylvaticum* as a pre-diapause host plant of the scarce fritillary (*Euphydryas maturna*) in Finland

**DOI:** 10.3897/BDJ.3.e5610

**Published:** 2015-07-17

**Authors:** Marko Nieminen

**Affiliations:** ‡ Metapopulation Research Centre, Department of Biosciences, University of Helsinki, Finland; § Faunatica Oy, Espoo, Finland

**Keywords:** *Euphydryas maturna*, Finland, Habitats Directive, host plant, *Melampyrum sylvaticum*, scarce fritillary

## Abstract

**Background:**

The scarce fritillary Euphydryas (Hypodryas) maturna (L.) is included in the Habitats Directive's Annexes II and IV(a). Therefore, it is crucially important to be able to define the habitat and breeding places of *E. maturna* in a correct and unbiased way.

**New information:**

Data on a previously unknown pre-diapause main host plant, the small cow-wheat (*Melampyrum sylvaticum* L.), of *Euphydryas maturna* in Finland is presented.

## Introduction

The scarce fritillary Euphydryas (Hypodryas) maturna (Linnaeus, 1758) is a high-profile species within the European Union, as it has been included in the Habitats Directive's (Council Directive 92/43/EEC of 21 May 1992 on the conservation of natural habitats and of wild fauna and flora) Annexes II and IV(a). Based on the Annex II, special conservation areas (*i.e.* Natura 2000 areas) need to be designated for *E. maturna*. The Annex IV lists species in need of strict protection, and those species and their breeding and resting places are protected by national legislation, which also applies to Finland. Therefore, the ability to define the habitat and breeding places of *E. maturna* in a correct and unbiased way is crucially important for both protecting the species effectively and not making uninformed administrative decisions which may be economically very costly. *Euphydryas maturna* is a wide-spread species in SW Finland, and it has been assessed as Least Concern by the IUCN criteria in Finland (Kaitila et al. 2010).

The species of the tribe Melitaeini, to which *E. maturna* belongs, feed mainly on plants containing secondary plant metabolites called iridoids (Bowers 1983, Wahlberg 2001). Iridoids are used for oviposition-plant selection, and as feeding stimulants and defensive chemicals by larvae (e.g. Bowers 1983, Nieminen et al. 2003, Wahlberg 2001). Larval host plants are the key part for the definition of breeding habitat for specialized herbivores such as *E. maturna*. Numerous plant species have been recorded as pre-diapause (Table 1) and/or post-diapause hosts of *E. maturna* throughout its range (see e.g. Wahlberg 1998, Dolek et al. 2013). In Finland, the common cow-wheat (*Melampyrum pratense* L.) has been recorded as the main host plant (Wahlberg 1998). Here, I present data on a previously unknown pre-diapause main host plant, the small cow-wheat (*Melampyrum sylvaticum* L.), of *Euphydryas maturna* in Finland.

## Materials and methods

Larval groups of *E. maturna* were systematically searched from an area of ca. 3.5 km^2^ within the municipalities of Sipoo and Pornainen in southern Finland (coordinates of the midpoint of the study area: 60.45072N, 25.30928E). All larval groups were georeferenced with GPS and photographed, and a sample of each host plant was collected for identification. Plant samples were identified by Henry Väre (Finnish Museum of Natural History, Helsinki). Fieldwork was made by MN and Kari Nupponen between August 27 and September 11 in 2014.

## Taxon treatments

### Euphydryas maturna

(Linnaeus, 1758)

#### Materials

- **Type status:** Other material. **Occurrence:** occurrenceRemarks: number of larval groups counted (with unknown number of larvae per group); recordedBy: Marko Nieminen; individualCount: 120; lifeStage: larva; **Taxon:** scientificName: Euphydryas maturna; order: Lepidoptera; family: Nymphalidae; genus: Euphydryas; specificEpithet: maturna; taxonRank: species; **Location:** country: Finland; stateProvince: Uusimaa; municipality: Sipoo; locality: Brusas; verbatimElevation: 60 m; verbatimCoordinates: 60°26.73'N 25°17.95'E; verbatimLatitude: 60°26.73'N; verbatimLongitude: 25°17.95'E; decimalLatitude: 60.4455; decimalLongitude: 25.2992; **Identification:** identifiedBy: Marko Nieminen; dateIdentified: 2014; **Event:** samplingProtocol: visual search; eventDate: 2014-08-27/09-11; **Record Level:** language: en; basisOfRecord: Photographed; source: marko.nieminen@faunatica.fi
- **Type status:** Other material. **Occurrence:** occurrenceRemarks: number of larval groups counted (with unknown number of larvae per group); recordedBy: Marko Nieminen; individualCount: 23; lifeStage: larva; **Taxon:** scientificName: Euphydryas maturna; order: Lepidoptera; family: Nymphalidae; genus: Euphydryas; specificEpithet: maturna; taxonRank: species; **Location:** country: Finland; stateProvince: Uusimaa; municipality: Pornainen; locality: Mäkelä; verbatimElevation: 60 m; verbatimCoordinates: 60°27.32'N 25°17.94'E; verbatimLatitude: 60°27.32'N; verbatimLongitude: 25°17.94'E; decimalLatitude: 60.4554; decimalLongitude: 25.2991; **Identification:** identifiedBy: Marko Nieminen; dateIdentified: 2014; **Event:** samplingProtocol: visual search; eventDate: 2014-08-27/09-11; **Record Level:** language: en; basisOfRecord: Photographed; source: marko.nieminen@faunatica.fi
- **Type status:** Other material. **Occurrence:** occurrenceRemarks: number of larval groups counted (with unknown number of larvae per group); recordedBy: Kari Nupponen; individualCount: 24; lifeStage: larva; **Taxon:** scientificName: Euphydryas maturna; order: Lepidoptera; family: Nymphalidae; genus: Euphydryas; specificEpithet: maturna; taxonRank: species; **Location:** country: Finland; stateProvince: Uusimaa; municipality: Pornainen; locality: Honkasenkalliot; verbatimElevation: 60 m; verbatimCoordinates: 60°26.51'N 25°19.27'E; verbatimLatitude: 60°26.51'N; verbatimLongitude: 25°19.27'E; decimalLatitude: 60.4419; decimalLongitude: 25.3211; **Identification:** identifiedBy: Kari Nupponen; dateIdentified: 2014; **Event:** samplingProtocol: visual search; eventDate: 2014-09-10/11; **Record Level:** language: en; basisOfRecord: Photographed; source: marko.nieminen@faunatica.fi

#### Ecology

**Figure 1.**
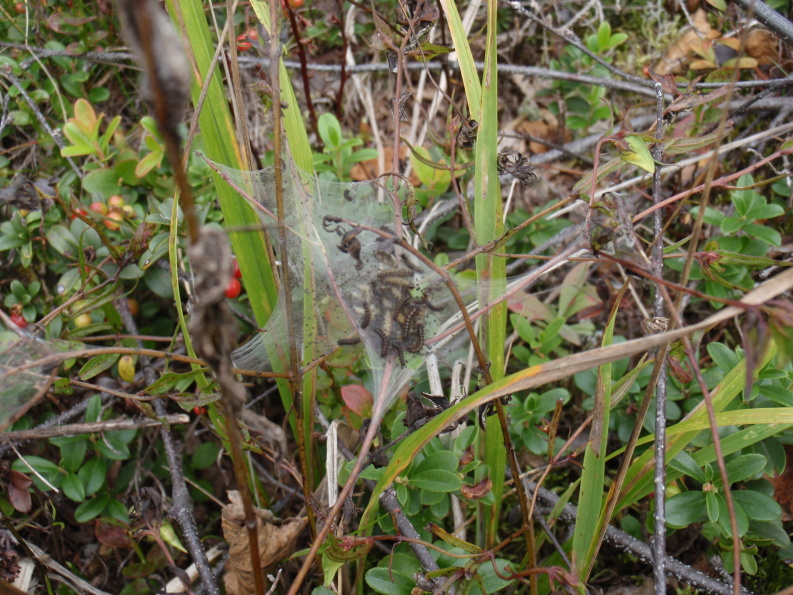
Larval web of *Euphydryas maturna* on *Melampyrum sylvaticum* in Sipoo, S Finland (November 2^nd^, 2014).

**Figure 2.**
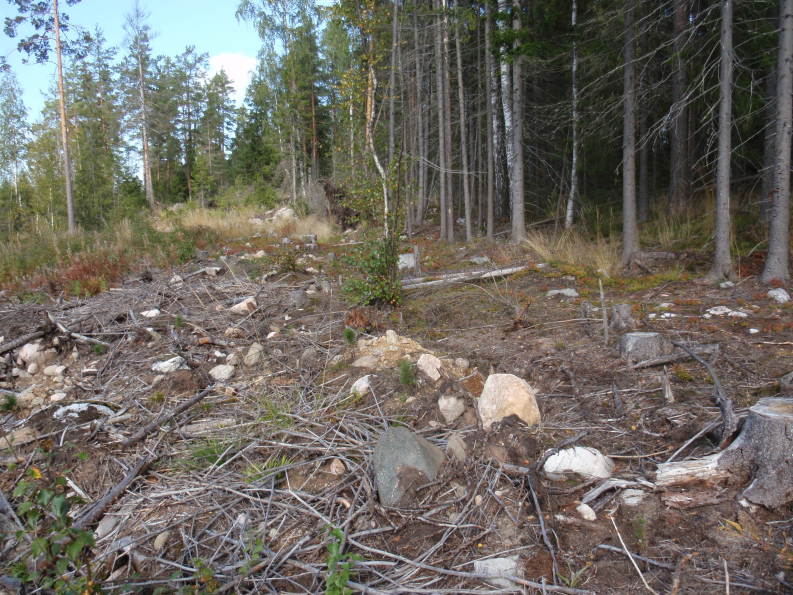
Clear-cut edge habitat of *Euphydryas maturna*. Clear-cut edges typically remain suitable for breeding for some years only until they become overgrown by tall grasses and tree seedlings.

**Figure 3.**
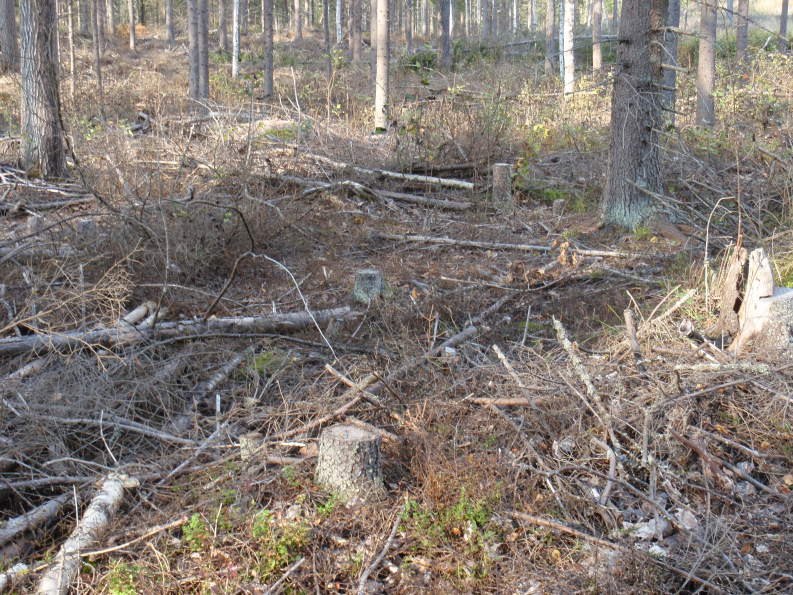
*Euphydryas maturna* habitat in a commercial, thinned spruce-dominated forest. Such habitats are probably suitable after thinning for several years.

**Figure 4.**
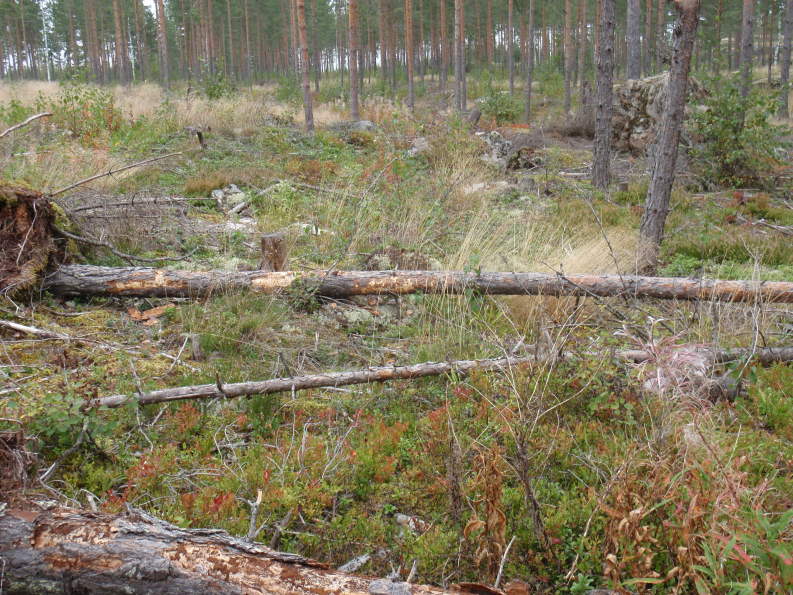
*Euphydryas maturna* habitat in a commercial, thinned pine-dominated forest with ca. 30-year old trees, and in a clear-cut edge. This kind of forest habitat is probably suitable after thinning for several years, but longer than spruce-dominated forests (Fig. 3). Also, edge habitats in these relatively dry habitats overgrow somewhat slower than in moister edges (Fig. 2).

**Figure 5.**
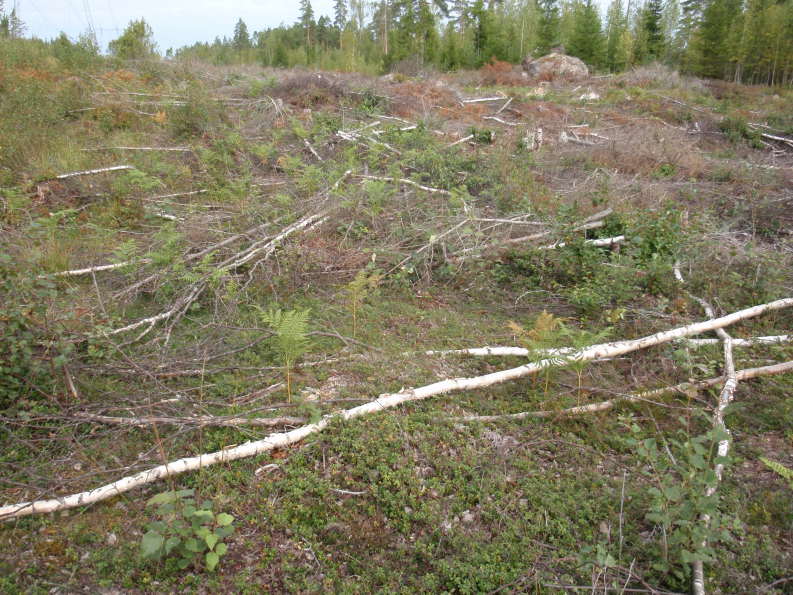
Powerline habitat of *Euphydryas maturna*. Vegetation under powerlines is kept open continuously, so powerline habitats may function both as breeding places and dispersal corridors.

Totally 167 larval groups were located, all on *Melampyrum* spp. (Fig. 1). In some cases, at least two original larval groups had probably merged. All larval groups were either in clear-cuts (usually close to the edges, and sometimes within the forest 0-5 m from the clear-cut [Figs 2, 4]), in thinned and light commercial forests (Figs 3, 4), or in open powerline corridors (Fig. 5).

Due to the dry conditions in July and August, many or even all host plants had withered especially in open rocky areas. Therefore, reliable identifications based on plant morphology were possible for 121 samples. Of the identified samples, 30 plants (25%) were *M. pratense* and 91 plants (75%) *M. sylvaticum*. In addition, three larval groups have been found in the same area in the autumn of 2013, all on *M. sylvaticum* (Jari-Pekka Kaitila, personal observations).

## Discussion

The regional host plant use of *E. maturna* is highly variable throughout its range (Table 1), but is apparently restricted to only a couple of preferred species used for oviposition within any particular region. For example, *Fraxinus* is the most regularly used oviposition-plant genus in the Central Europe (e.g. Cizek and Konvicka 2005, Levente 2005, Freese et al. 2006, Dolek et al. 2013), whereas lower plants such as *Veronica longifolia* are often used in the eastern areas (e.g. Korshunov and Gorbunov 1995, Gorbunov and Kosterin 2007). However, it seems common that the post-diapause larvae feed on a wider spectrum of host plants than are used for oviposition (Gorbunov and Kosterin 2007, Dolek et al. 2013), for example *Plantago lanceolata* is frequently used after diapause in Austria (Freese et al. 2006). There may be regional differences in preference also within the Finnish range, as all females observed during their search for oviposition-plants ignored *M. sylvaticum* in a study performed about 200 km to the northeast of this study area (Wahlberg 1998). Moreover, the importance of other host plants than *M. pratense* and *M. sylvaticum* still remain uncertain throughout Finland.

The use of *M. sylvaticum* as a host plant (Fig. 1) considerably increases both the suitable breeding area of *E. maturna* and the amount of resources available for it. In the study area, the increase in both of these variables must be manyfold, but remains to be quantified. *Melampyrum pratense* is much more vulnerable to desiccation and withering because it grows in drier sites than *M. sylvaticum*. The ability to use both of these *Melampyrum* species is extremely important in dry summers such as 2014, when more than 90% of host plants had withered in several sites. That high rate of dry host plants has likely increased mortality of groups of small larvae and may also decrease overwintering success due to starvation of larvae, which are common phenomena in another larval group-forming species *Melitaea cinxia* in Finland (e.g. Kuussaari et al. 2004).

Some leaves of *Vaccinium myrtillus* had been eaten within some larval webs. Even though I could not confirm that *E. maturna* larvae had eaten them, it is, however, likely because other herbivorous larvae were not observed and *Melampyrum* individuals were almost completely dry in and around these larval nests. Therefore, larvae may have used *V. myrtillus* to rescue themselves from starvation. The same explanation may apply to the odd observations of larvae feeding on e.g. *Fagus*, *Populus* and *Salix* regularly referred to in the literature (e.g. Korshunov and Gorbunov 1995, Gorbunov and Kosterin 2007, Dolek et al. 2013). A further explanation for 'odd' host records is that the actual host plants often become consumed completely leaving only non-host plants visible among and next to larval webs.

## Supplementary Material

XML Treatment for Euphydryas maturna

## Figures and Tables

**Table 1.** Records of host plants used for oviposition by *Euphydryas maturna* females and/or for feeding by pre-diapause larvae.

Species	Locations	References
*Fagus sylvatica*	Europe	Dolek et al. 2013
*Fraxinus angustifolia*	Carpathian Basin and SE Europe, Hungary	Dolek et al. 2013, Rákosy et al. 2012
*Fraxinus excelsior*	Austria, Czech Republic, Germany, Hungary, Italy, Russia, Sweden	Dolek et al. 2013, Eliasson 1991, Freese et al. 2006, Konvicka et al. 2005, Levente 2005, Rákosy et al. 2012, Tuzov et al. 2000
*Fraxinus ornus*	Carpathian Basin and SE Europe	Dolek et al. 2013, Rákosy et al. 2012
*Ligustrum vulgare*	Czech Republic, Germany, Hungary	Dolek et al. 2013, Freese et al. 2006, Konvicka et al. 2005, Rákosy et al. 2012
* Lonicera *	Russia	Tuzov et al. 2000
*Melampyrum sylvaticum*	Finland	This study
*Melampyrum pratense*	Finland	Wahlberg 1998
*Plantago lanceolata*	Komi Republic	Gorbunov and Kosterin 2007
*Populus alba*	Russia	Tuzov et al. 2000
*Populus tremula*	Europe, Russia	Dolek et al. 2013, Tuzov et al. 2000
*Salix caprea / Salix*	Europe, Russia	Dolek et al. 2013, Tuzov et al. 2000
* Spiraea *	Russia	Tuzov et al. 2000
*Syringa vulgaris*	Russia, Sweden	Dolek et al. 2013, Eliasson and Shaw 2003, Tuzov et al. 2000
*Veronica longifolia*	Finland, Komi Republic, Krasnoyarsk area, Omsk area, Novosibirsk Province	Gorbunov and Kosterin 2007, Wahlberg 1998
*Viburnum opulus*	Finland, Germany, Sweden	Dolek et al. 2013, Eliasson 1991, Wahlberg 1998
*Viola arvensis*	Komi Republic	Gorbunov and Kosterin 2007
*Viola canina*	Komi Republic	Gorbunov and Kosterin 2007
